# Among classic myeloproliferative neoplasms, essential thrombocythemia is associated with the greatest risk of venous thromboembolism during COVID-19

**DOI:** 10.1038/s41408-021-00417-3

**Published:** 2021-02-04

**Authors:** Tiziano Barbui, Valerio De Stefano, Alberto Alvarez-Larran, Alessandra Iurlo, Arianna Masciulli, Alessandra Carobbio, Arianna Ghirardi, Alberto Ferrari, Valeria Cancelli, Elena Maria Elli, Marcio Miguel Andrade-Campos, Mercedes Gasior Kabat, Jean-Jaques Kiladjian, Francesca Palandri, Giulia Benevolo, Valentin Garcia-Gutierrez, Maria Laura Fox, Maria Angeles Foncillas, Carmen Montoya Morcillo, Elisa Rumi, Santiago Osorio, Petros Papadopoulos, Massimiliano Bonifacio, Keina Susana Quiroz Cervantes, Miguel Sagues Serrano, Gonzalo Carreno-Tarragona, Marta Anna Sobas, Francesca Lunghi, Andrea Patriarca, Begoña Navas Elorza, Anna Angona, Elena Magro Mazo, Steffen Koschmieder, Giuseppe Carli, Beatriz Cuevas, Juan Carlos Hernandez-Boluda, Emma Lopez Abadia, Blanca Xicoy Cirici, Paola Guglielmelli, Marta Garrote, Daniele Cattaneo, Rosa Daffini, Fabrizio Cavalca, Beatriz Bellosillo, Lina Benajiba, Natalia Curto-Garcia, Marta Bellini, Silvia Betti, Claire Harrison, Alessandro Rambaldi, Alessandro Maria Vannucchi

**Affiliations:** 1 FROM Research Foundation, Papa Giovanni XXIII Hospital, Bergamo, Italy; 2 Section of Hematology, Department of Radiological and Hematological Sciences, Catholic University, Fondazione Policlinico “A. Gemelli” IRCCS, Rome, Italy; 3 Hospital Clinic de Barcelona, Barcelona, Spain; 4 Hematology Division, Foundation IRCCS Ca’ Granda Ospedale Maggiore Policlinico, Milan, Italy; 5 Spedali Civili, Brescia, Italy; 6 Hematology Division and Bone Marrow Transplant, San Gerardo Hospital, ASST Monza, Monza, Italy; 7 Hospital del Mar – IMIM, Barcelona, Spain; 8 Hospital Universitario la Paz, Madrid, Spain; 9 Hospital Saint-Louis, Paris, France; 10 Azienda Ospedaliero-Universitaria di Bologna, Via Albertoni 15, Bologna, Italia; 11 AOU Città della Salute e della Scienza di Torino, Torino, Italy; 12 Hospital Ramon y Cajal, IRYCIS, Madrid, Spain; 13 Department of Hematology, Vall d’Hebron Institute of Oncology (VHIO), Vall d’Hebron Hospital Universitari, Vall d’Hebron Barcelona Hospital Campus, C/ Natzaret, 115-117, 08035 Barcelona, Spain; 14 Hospital Universitario Infanta Leonor, Madrid, Spain; 15 Hospital General Universitario de Albacete, Albacete, Spain; 16 Department of molecular medicine, University of Pavia, Pavia, Italy; 17 Hospital Gregorio Maranon, Madrid, Spain; 18 Hospital Clinico San Carlos, Madrid, Spain; 19 Ospedale Policlinico “G.B. Rossi”, Borgo Roma, Verona, Italy; 20 Hospital Universitario de Mostoles, Madrid, Spain; 21 ICO L’Hospitalet-Hospital Moises Broggi, Sant Joan Despì, Barcelona, Spain; 22 Hospital Universitario 12 de Octubre, Madrid, Spain; 23 Department of Hematology, Blood Neoplasms and Bone Marrow Transplantation, Wroclaw Medical University, Wrocław, Poland; 24 IRCCS Ospedale San Raffaele, Milano, Italy; 25 AOU Maggiore della Carità, Novara, Italy; 26 Hospital Moncloa, Madrid, Spain; 27 ICO Girona Hospital Josep Trueta, Girona, Spain; 28 Hospital Universitario Principe de Asturias, Alcalà de Henares, Madrid, Spain; 29 Department of Hematology, Oncology, Hemostaseology, and Stem Cell Transplantation, Faculty of Medicine, RWTH Aachen University, Aachen, Germany; 30 Ospedale San Bortolo, Vicenza, Italy; 31 Hospital Universitario de Burgos, Burgos, Spain; 32 Hospital Clinico Universitario, INCLIVA, Valencia, Spain; 33 Hospital General de Elche, Elche, Alicante, Spain; 34 Institut Català d’Oncologia-Hospital Germans Trias i Pujol, Joseo Carreras Leukemia Research Institute, Badalona (Barcelona) Spain, Universitat Autònoma de Barcelona, Barcelona, Spain; 35 Center Research and Innovation of Myeloproliferative Neoplasms (CRIMM), Department of Experimental and Clinical Medicine, Azienda Ospedaliera Universitaria Careggi, University of Florence, Florence, Italy; 36 Guy’s and St. Thomas’ NHS Foundation Trust, London, UK; 37 ASST Papa Giovanni XXIII, Bergamo, Italy; 38 Università degli Studi di Milano, Milano, Italy

**Keywords:** Risk factors, Myeloproliferative disease, Infectious diseases

## Abstract

In a multicenter European retrospective study including 162 patients with COVID-19 occurring in essential thrombocythemia (ET, *n* = 48), polycythemia vera (PV, *n* = 42), myelofibrosis (MF, *n* = 56), and prefibrotic myelofibrosis (pre-PMF, *n* = 16), 15 major thromboses (3 arterial and 12 venous) were registered in 14 patients, of whom all, but one, were receiving LMW-heparin prophylaxis. After adjustment for the competing risk of death, the cumulative incidence of arterial and venous thromboembolic events (VTE) reached 8.5% after 60 days follow-up. Of note, 8 of 12 VTE were seen in ET. Interestingly, at COVID-19 diagnosis, MPN patients had significantly lower platelet count (*p* < 0.0001) than in the pre-COVID last follow-up.This decline was remarkably higher in ET (−23.3%, *p* < 0.0001) than in PV (−16.4%, *p* = 0.1730) and was associated with higher mortality rate (*p* = 0.0010) for pneumonia. The effects of possible predictors of thrombosis, selected from those clinically relevant and statistically significant in univariate analysis, were examined in a multivariate model. Independent risk factors were transfer to ICU (SHR = 3.73, *p* = 0.029), neutrophil/lymphocyte ratio (SHR = 1.1, *p* = 0.001) and ET phenotype (SHR = 4.37, *p* = 0.006). The enhanced susceptibility to ET-associated VTE and the associated higher mortality for pneumonia may recognize a common biological plausibility and deserve to be delved to tailor new antithrombotic regimens including antiplatelet drugs.

## Introduction

Coronavirus-2 (SARS-CoV-2) infection, when presenting as coronavirus disease 2019 (COVID-19), mainly affects the respiratory system. However, multiple organ damage, involving the brain, heart, kidney, and vascular system are being reported with increasing frequency, and contribute to the high mortality rate. COVID-19 is characterized by the occurrence of venous thromboembolism (VTE) and/or arterial thrombosis, as well as occlusions in the microcirculation, leading to severe morbidity and mortality^1–3^. A possible mechanism of this systemic damage may be that coronavirus attacks the human body through the angiotensin-converting enzyme 2 (ACE2), which is distributed over blood vessels and in various organs^4,5^.

The incidence rate of thromboembolic events is variable and largely depends on whether patients were specifically screened for the presence of thrombosis and hospitalized in the intensive care unit (ICU) rather than in regular wards^6^. In hospitalized COVID-19 patients, the overall incidence of these events was estimated 22.7% in patients transferred to ICU and 7.9% in general wards^6^. On the basis of autopsy findings, extensive thrombosis of pulmonary microvascular circulation has been consistently documented^7^ and the concept of “in situ” pulmonary thrombosis associated with respiratory worsening and eventually death, was proposed^8,9^.

VTE and arterial thrombosis may be a major concern in some categories of patients with SARS-CoV-2 infection, such as the ones with generic cardiovascular risk factors, including hypertension, obesity, diabetes, or cardiovascular and chronic respiratory disease, and cancer. Patients with myeloproliferative neoplasms (MPN), who are constitutively prone to develop thrombotic complications, might be amongst the most vulnerable to these events. Patients with essential thrombocythemia (ET), polycythemia vera (PV), and myelofibrosis (MF) suffer from a 2–4-fold higher incidence of arterial and venous thrombosis than the general population^10,11^, and also the frequency of spontaneous or drug-related bleeding is also higher than in general population^12^. Therefore, knowing whether the rate of thrombotic and hemorrhagic events in the course of COVID-19 is higher in these patients than in the general COVID-19 population and identifying associated risk factors, could be of great benefit in individualizing prophylaxis and antithrombotic therapy.

Based on these premises and given that the prevalence of MPN is relatively low^13,14^, we launched an international multicenter study collecting data in ET, PV, and MF patients with COVID-19, recruited in specialized hematology European centers, during the first wave of the pandemic. The primary goal was to evaluate the incidence of thrombosis and bleeding and to identify associated predictors.

## Subjects and methods

MPN-COVID (ClinicalTrials.gov identifier: NCT04385160) is a multicenter, retrospective, cohort study that involved 38 hematology units in Europe, on behalf of European LeukemiaNet (ELN) and with the endorsement of the European Hematology Association (EHA) and GEMFIN Spanish network.

The sponsor of the study is FROM—Fondazione per la Ricerca Ospedale di Bergamo at Papa Giovanni XXIII hospital in Bergamo (Italy), which obtained national approval for conducting the study in Italy by the ethical committee of the Spallanzani Hospital (approval number: 77-2020), in Rome (Italy); all other participating centers got the approval by their respective local ethical committees and institutional review boards.

Eligible patients were consecutive adults (aged ≥ 18 years) with WHO-2016 diagnosis of MPN and SARS CoV-2 infection detected between February 15 and May 31, 2020. The minimum follow-up time was 1 month; the study was closed on June 30, 2020. An amendment to the original protocol (version no. 2, 25/06/2020) has been approved to extend the follow-up study period by an additional 6 months after the last study visit for survivors.

Inclusion criteria included hospitalized COVID-19 cases ascertained by a positive real-time reverse transcriptase polymerase chain reaction from nasopharyngeal swab; however, given the exceptional situation of emergency determined by pandemic crisis, also home-treated patients unable to swab were included, when the presence of pneumonia and symptoms were highly suggestive for SARS CoV-2 infection (i.e., fever, cough, oxygen saturation (O_2_ saturation) ≤ 93% at rest, dyspnea, diarrhea). For the purposes of this analysis, only patients with non-missing information regarding assessment of thrombosis were included.

Written informed consent of participants was collected whenever possible according to each Country regulation.

Data collection was centralized at FROM and performed through a dedicated Electronic Data Capture (EDC) system supporting electronic case report forms (e-CRFs) set up on purpose. Data on demographic and clinical characteristics, laboratory parameters, and drug exposure were collected at relevant time points, namely (i) last MPN follow-up visit before COVID-19 onset; (ii) COVID-19 diagnosis; (iii) MPN follow-up after COVID-19; and (iv) last visit.

All nasopharyngeal swabs for COVID-19 diagnosis were managed according to national recommendations and COVID-19 disease was assessed according to current WHO guidance^15^. Respiratory disease severity at COVID-19 diagnosis was categorized based on oxygen support needs, as non-intensive (including low-flow oxygen, Venturi mask, non-rebreather masks, continuous positive airway pressure [CPAP], non-invasive ventilation [NIV]) and intensive (whether in need of either endotracheal intubation, or invasive mechanical ventilation, or extra corporeal membrane oxygenation [ECMO]).

The primary outcome was the incidence of pulmonary embolism (PE) with or without deep vein thrombosis (DVT) of the legs, which had to be confirmed by imaging (ultrasounds and computed tomography [CT] angiography). Presumed PEs were cases unable to be confirmed by imaging due to the practical limitations imposed by the severity of infection and were also registered if they were defined by sudden increase of either D-Dimer in patients with rapid worsening of respiration and/or increase of oxygen need.

Secondary outcomes were the occurrence of any other major thrombosis, major bleeding and death. All outcomes were measured during hospitalization for COVID-19 or assessed by the family doctor for patients managed at home. Myocardial infarction, stroke, and peripheral arterial thrombosis were diagnosed by objective tests including electrocardiographic changes, cerebral CT scan, and angiography, respectively. For the definition and assessment of thrombotic and hemorrhagic events, the International Statistical Classification of Diseases and Related Health Problems 10th Revision (ICD-10)-WHO current version^16^ were used.

The use of low molecular weight heparin (LMWH) as antithrombotic prophylaxis was recorded, defining the dose as prophylactic low-dose (4000–6000 IU sc fixed-dose q 24 h) or therapeutic dose (100 IU/kg bw sc q 12 h). LMWH doses higher than the prophylactic ones but not reaching those of therapeutic level were defined as intermediate dose.

Potential risk factors for primary and secondary endpoints were investigated, including patient comorbidities, MPN type and status, MPN-directed therapies, laboratory parameters at COVID-19 diagnosis, COVID-19 severity, and treatments. Results concerning mortality rate in this series were previously reported^17^.

### Statistical analysis

An initial sample size of at least 80 patients was estimated to detect a proportion of PE of 0.35 vs. 0.20, as reported in non-MPN COVID-19 cases (alpha = 0.05; beta = 0.20).

Continuous variables are expressed as median (IQR) and the Kruskal–Wallis test was used to compare MPN phenotypes. Categorical variables are presented as frequencies and percentages, and were analyzed using the *χ*² test or Fisher’s exact test, as appropriate.

Cumulative incidence function (CIF) for death, thrombosis, and bleeding were estimated considering death as competing event. Univariate analysis testing the association of each explanatory variables and thrombosis were performed using the Mann–Whitney *U* test, or *χ*² test, or Fisher’s exact test, as appropriate. Multivariable Fine and Gray regression model was finally applied to find independent predictors of thrombosis among those clinically relevant and significantly associated in univariate analysis, treating death as competing event.

Two-sided *P*-values of 0.05 or less were considered to indicate statistical significance. All analyses were performed using Stata (StataCorp, College Station, TX) statistical software, release 16.1.

## Results

### Description of thrombotic and major bleeding events

In Table 1, we report the frequency and types of thrombosis and bleeding observed over a median of 50.5 days of follow-up (IQR: 16.0–69.0) in 162 patients with MPN fulfilling eligibility criteria defined for this analysis. During the acute phase of infection, 19 patients experienced 22 thrombo-hemorrhagic events at home (*n* = 1/40, 2.5%), or during hospitalization in regular wards (*n* = 13/105, 12.4%) or in ICU (*n* = 5/17, 29.4%) (*p* = 0.016). Overall, only three arterial thrombosis were documented (1.9%), whereas the majority were VTE (*n* = 12, 7.4%), detected with a significantly higher frequency in ET patients (*n* = 8/48, 16.7%) than in PV (*n* = 2/42, 4.8%) or MF (*n* = 2/56, 3.6%) (p = 0.031). In ET, 7 of 8 VTE (87.5%) were PE, of which one concomitant with DVT of the legs. Two additional PE were seen in patients with PV and MF each. All but two patients with VTE were receiving anticoagulation with low or intermediate doses (*n* = 5/10, 50%) or therapeutic doses (*n* = 5/10, 50%) of LMWH. Mortality was recorded in 7 of 14 (50%) patients with thrombosis, of which one was attributed to acute myocardial infarction, while the other 6 were not considered to be related to the previous vascular event; causes of death were indistinguishable from other concomitant conditions, such as multi-organ failure (MOF) (*n* = 4), pneumonia (*n* = 1), or unknown reason (*n* = 1).

**Table 1:** Types of thrombosis and bleeding, by MPN phenotypes.

	Total	ET	PV	MF	Pre-PMF	*p*-value
	*N* = 162	*N* = 48	*N* = 42	*N* = 56	*N* = 16	
*Thrombosis*						
Pts with thrombosis, *n* (%)	14 (8.6%)	8 (16.7%)	2 (4.8%)	3 (5.4%)	1 (6.3%)	0.13
N thrombosis, *n* (%)	15 (10.0%)	9 (18.8%)	2 (4.8%)	3 (5.4%)	1 (6.3%)	0.35
Arterial, *n* (%)	3 (1.9%)	1 (2.1%)	0 (0.0%)	1 (1.8%)	1 (6.3%)	0.47
AMI	1	0		1	0	
Stroke	1	1		0	0	
PAT	1	0		0	1	
Venous, *n* (%)	12 (7.4%)	8 (16.7%)	2 (4.8%)	2 (3.6%)	0 (0.0%)	0.031
PE^a^	10	6	2	2		
DVT+PE^a^	1	1	0	0		
SVT	1	1	0	0		
Time to any thrombosis, days, median (IQR)	11.5 (4.0–25.0)	7.0 (2.5–11.5)	8.0 (1.0–15.0)	25.0 (16.0–32.0)	28.0 (28.0–28.0)	0.27
Time to PE, days, median (IQR)	8.0 (0.0–14.0)	4.0 (0.0–8.0)	7.0 (0.0–14.0)	27.5 (24.0–31.0)		0.097
*Bleeding*						
Pts with bleeding, *n* (%)	7 (4.3%)	1 (2.1%)	2 (4.9%)	4 (7.1%)	0 (0.0%)	0.51
Sites						0.20
Gastrointestinal	3	0	0	3		
Muscle	2	1	1	0		
Hemoptysis	2	0	1	1		
Trasfusion need, *n* (%)	5 (3.1%)	1 (2.1%)	0 (0.0%)	4 (7.1%)		0.030
Time to bleeding, days, median (IQR)	16.0 (13.0–24.0)	14.0 (14.0–14.0)	23.0 (16.0–30.0)	17.5 (6.5–23.0)		0.50

*ET* essential thrombocythemia, *PV* polycythemia vera, MF myelofibrosis, *pre-PMF* prefibrotic myelofibrosis, *Pts* patients, *AMI* acute myocardial infarction, *PAT* pheripheral arterial thrombosis, *PE* pulmonary embolism, *DVT* deep vein thrombosis of the legs, *SVT* superficial vein thrombosis, *IQR* interquartile range.

^a^PE diagnosis confirmed by imaging *n* = 8, not confirmed *n* = 3.

Seven patients of 162 (4.3%) developed major bleeding, in particular in MF, where all the four hemorrhagic episodes required blood transfusions. These events were diagnosed starting from the second week after the onset of the SARS-CoV-2 infection and occurred mainly in patients admitted to regular ward (*n* = 6/7, 85.7%) and in one case in ICU (14.3%). Details of bleeding in the seven patients are illustrated in Table 2. We note that most of the bleeding events occurred in patients over the age of 70 years with MF and affected the gastrointestinal tract in three cases. All but two patients were receiving anticoagulant treatment: four were on LMWH (low-dose *n* = 3, therapeutic dose *n* = 1) and one was on apixaban. Only patient #1 with MF (Table 2) had an activated partial thromboplastin time (aPTT) prolongation and a moderate thrombocytopenia despite the absence of heparin prophylaxis. Severe thrombocytopenia < 30 × 10^9^/L was noted in three patients (#5, #6, and #7), two of them receiving low-dose LMWH. All bleeding episodes but one occurred in patients admitted to regular wards.

**Table 2:** Bleeding events.

Pt. no.	Sex	Age, yr	MPN	Disposition	Site of bleeding	Days from COVID-19 diagnosis	Anticoagulation	Transfusion need	Plt (×10^9^/L)^a^	aPTT (s)^a^	Fibrinogen (mg/dL)^a^
#1	M	89.6	MF	Regular ward	Gastrointestinal	0	No	Yes	97	76	469
#2	F	60.0	ET	Regular ward	Muscle	14	Enoxaparin, terapeutic dose	Yes	461	31.9	220
#3	F	70.6	PV	Regular ward	Muscle	16	Enoxaparin, low dose	No	244	28.6	355
#4	F	80.6	PV	Regular ward	Hemoptsysis	30	Apixaban	No	NA	NA	NA
#5	M	78.9	MF	Regular ward	Gastrointestinal	13	No	Yes	16	NA	NA
#6	M	65.2	MF	ICU	Hemoptsysis	24	Parnaparin, low dose	Yes	28	34.3	670
#7	F	75.4	MF	Regular ward	Gastrointestinal	22	Enoxaparin, low dose	Yes	29	24	96

*Pt* patient, *y* year, *Plt* platelets count, *aPTT* activated partial thromboplastin time, *M* male, *F* female, *ET* essential thrombocythemia, *PV* polycythemia vera, *MF* myelofibrosis, *ICU* intensive care unit, *NA* not available.

^a^Measured at bleeding event.

### Cumulative incidence of thrombosis and bleeding

**Fig. 1:**
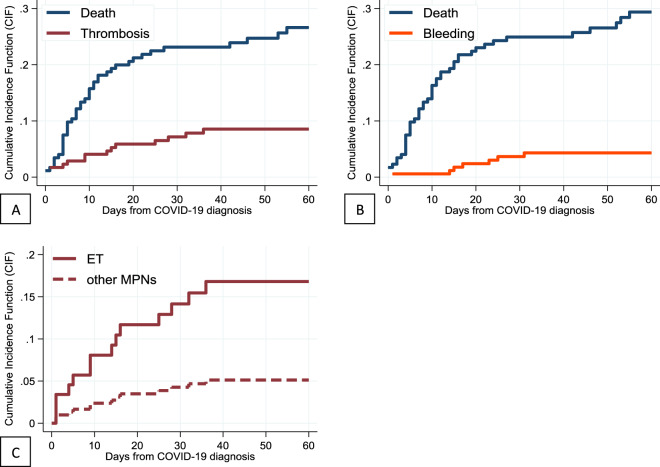
Cumulative incidence of thrombosis and bleeding. Cumulative Incidence Function (CIF) of thrombosis overall (**A**) and by MPN phenotypes (**C**), and bleeding (**B**), considering death as competing event.

Figure 1a reports CIF curves for the competing risks of death and thrombosis. At 60 days, mortality reached 26.6% (95% CI: 4.9–13.4%). Most of the fatal events occurred within the first 15 days (20%); of these, 7 in patients experiencing major thrombosis (see above) and subsequently the curve grew more slowly until 30 days, becoming almost stable afterwards. Adjusting for the competing risk of dying before the event, probability of total thrombosis progressively increased in the first month and reached 8.5% after 60 days follow. The cumulative incidence rate at 10, 20, 30 days was 4.1%, 5.9% and 7.1%, respectively. In COVID-19 patients followed at home or in regular ward (*n* = 40, 25% and *n* = 105, 65%, respectively), the rate was 1.0% and 2.8%, respectively, whereas it reached 18.4% in the 17 (10% of the entire cohort) ICU patients.

The overall major bleeding rate was 4.3% (95% CI: 1.9–8.2%) and the 7 events occurring in 7 patients accounted for a cumulative incidence rate of 0.6%, 2.4% and 3.6% at 10, 20, 30 days, respectively (Fig. 1b).

Fatal events were reported in 41 of 162 patients (25.3%), and the two most frequently reported were pneumonia (*n* = 15, 37%) and MOF (*n* = 17, 41%), being the remaining unknown or unspecified causes (*n* = 9, 22%). Note that among the 11 deaths that occurred in ET patients, pneumonia was more frequent (*n* = 6, 55%) than in PV (*n* = 2/6, 33%) and MF (*n* = 6/21, 29%) while there was no difference between fatalities from MOF in ET (*n* = 5/11, 45%) and PV (*n* = 3/6, 50%). Remarkably, ET patients with thromboses vs. the ones without these events had a survival probability of 53.6% (95% CI: 23.3–76.6%) and 75.6% (95% CI: 67.5–82.0%), respectively (*p* = 0.052) (supplementary Fig. 1S).

### Univariate and multivariate analysis for thrombosis

MPN patients’ characteristics stratified by thrombosis are presented in Table 3. Gender, age and MPN type distribution were comparable in the two groups, with the exception of a significantly higher proportion of thrombosis in ET patients (*p* = 0.018). We did not find any correlation between driver mutations and the incidence of thrombosis. In particular, no difference was found in patients with or without thrombosis according to the presence of *JAK2*V617F, even if stratified by MPN phenotypes. Of note, ET, compared to the other phenotypes (Supplementary Table 1S), showed no difference in terms of COVID-19 severity, as defined by age, patient managed at home, admitted to regular ward or ICU, and inflammatory markers, with the exception of higher platelet counts at COVID-19 diagnosis (*p* = 0.012).

**Table 3:** Univariate analysis for thrombosis.

	Total	No thrombosis	Thrombosis	*p*-value
	*N* = 162	*N* = 148	*N* = 14	
Sex*, n (%)*				1.00
Female	66 (40.7%)	60 (40.5%)	6 (42.9%)	
Male	96 (59.3%)	88 (59.5%)	8 (57.1%)	
Age (years), median (IQR)	70.6 (60.0–79.9)	70.6 (60.0–79.9)	70.6 (60.0–80.0)	0.91
<60 years, *n* (%)	40 (24.8%)	37 (25.2%)	3 (21.4%)	1.00
60–70 years, *n* (%)	36 (22.4%)	33 (22.4%)	3 (21.4%)	
>70 years, *n* (%)	85 (52.8%)	77 (52.4%)	8 (57.1%)	
MPN diagnosis, *n* (%)				0.17
ET	48 (29.6%)	40 (27.0%)	8 (57.1%)	0.018
PV	42 (25.9%)	40 (27.0%)	2 (14.3%)	0.30
MF	56 (34.6%)	53 (35.8%)	3 (21.4%)	0.28
Pre-PMF	16 (9.9%)	15 (10.1%)	1 (7.1%)	0.72
JAK2V617F, *n* (%)	112 (70.9%)	103 (71.5%)	9 (64.3%)	0.57
CALR, *n* (%)	26 (29.5%)	23 (28.7%)	3 (37.5%)	0.61
MPL, *n* (%)	5 (6.0%)	4 (5.2%)	1 (16.7%)	0.26
Previous thrombosis, *n* (%)	23 (14.4%)	20 (13.7%)	3 (21.4%)	0.43
Previous bleeding, *n* (%)	12 (7.5%)	12 (8.2%)	0 (0.0%)	0.26
MPN duration before COVID-19 onset (years), median (IQR)	5.9 (2.9–10.8)	5.9 (2.6–10.4)	5.9 (4.1–10.9)	0.60
*Blood values before COVID-19 diagnosis*^a^, *median (IQR)*				
Hemoglobin, g/dL	13.0 (11.4–14.2)	12.9 (11.3–14.3)	13.1 (12.2–14.0)	0.77
White blood cells, ×10^9^/L	7.2 (5.4–10.3)	7.2 (5.5–10.1)	6.5 (5.2–12.5)	0.66
Platelets, ×10^9^/L	326.0 (218.0–477.0)	320.0 (214.5–466.0)	369.5 (288.0–550.0)	0.29
*MPN-directed therapy before COVID-19 diagnosis*^a^, *n* (%)				
Hydroxyurea	73 (45.1%)	66 (44.6%)	7 (50.0%)	0.70
Discontinued after COVID-19^b^	9/46 (20%)	7/39 (18%)	2/7 (28%)	0.61
Ruxolitinib	40 (24.7%)	37 (25.0%)	3 (21.4%)	0.77
Discontinued after COVID-19	9/40 (23%)	8/37 (22%)	1/3 (33%)	0.55
Anagrelide	8 (4.9%)	6 (4.1%)	2 (14.3%)	0.091
Interferon	4 (2.5%)	4 (2.7%)	0 (0.0%)	0.53
Other cytoreductive drugs	5 (3.1%)	5 (3.4%)	0 (0.0%)	0.48
ASA	94 (58.0%)	83 (56.1%)	11 (78.6%)	0.10
*Patient disposition*, *n* (%)				0.010
Home	40 (24.8%)	39 (26.5%)	1 (7.1%)	
Regular ward	104 (64.6%)	96 (65.3%)	8 (57.1%)	
ICU	17 (10.6%)	12 (8.2%)	5 (35.7%)	
*Main symptoms*, *n* (%)				
Fever	130 (80.2%)	118 (79.7%)	12 (85.7%)	0.59
Dispnea	89 (54.9%)	79 (53.4%)	10 (71.4%)	0.19
Gastrointestinal	19 (11.7%)	19 (12.8%)	0 (0.0%)	0.15
*Comorbidities*, *n* (%)				
Cerebrovascular disease	21 (13.0%)	19 (12.9%)	2 (14.3%)	0.89
Chronic dialysis/Kidney disease	15 (9.3%)	14 (9.5%)	1 (7.1%)	0.77
Chronic heart failure	22 (13.8%)	20 (13.6%)	2 (15.4%)	0.86
COPD	22 (13.7%)	20 (13.6%)	2 (14.3%)	0.94
Current/former tobacco smoker	33 (23.2%)	31 (24.2%)	2 (14.3%)	0.40
Hyperlipidemia	45 (29.0%)	41 (28.9%)	4 (30.8%)	0.89
Hypertension	97 (61.4%)	91 (63.2%)	6 (42.9%)	0.14
Antihypertensives use	93 (60.8%)	89 (64.0%)	4 (28.6%)	0.010
ACE inhibitors/ARBs	54 (36.7%)	53 (39.6%)	1 (7.7%)	0.023
Other	33 (21.6%)	31 (22.3%)	2 (14.3%)	0.49
Diabetes mellitus	19 (11.9%)	18 (12.3%)	1 (7.7%)	0.62
O_2_ saturation (%), median (IQR)	93.0 (88.0–96.0)	93.0 (88.0–96.0)	94.0 (90.0–96.0)	0.81
*COVID-19-directed drugs, n (%)*				
Steroid	41 (27.2%)	34 (24.8%)	7 (50.0%)	0.044
Antibiotic	104 (68.9%)	91 (66.4%)	13 (92.9%)	0.042
Hydroxychloroquine	93 (59.6%)	84 (59.2%)	9 (64.3%)	0.71
Antiviral	54 (35.1%)	48 (34.3%)	6 (42.9%)	0.52
Lopinavir/Ritonavir	44 (88.0%)	38 (86.4%)	6 (100.0%)	1.00
Other	6 (12.0%)	6 (13.6%)	0 (0.0%)	
Experimental	17 (10.7%)	14 (9.7%)	3 (21.4%)	0.17
Tocilizumab	13 (76.5%)	10 (71.4%)	3 (100.0%)	1.00
Ruxolitinib	2 (11.8%)	2 (14.3%)	0 (0.0%)	
Other	2 (11.8%)	2 (14.3%)	0 (0.0%)	
Antithrombotic	88 (56.8%)	76 (53.9%)	12 (85.7%)	0.022
LMWH	84 (54.2%)	72 (51.1%)	12 (85.7%)	0.013
Low-intermediate dose	62 (79.5%)	56 (74.8%)	6 (50.0%)	0.34
Therapeutic dose	16 (20.5%)	10 (15.2%)	6 (50.0%)	
*Laboratory parameters at COVID-19 diagnosis, median (IQR)*				
Hemoglobin, g/dL	12.4 (10.0–13.5)	12.3 (9.9–13.5)	12.9 (11.4–13.8)	0.37
White blood cells, ×10^9^/L	6.6 (4.7–10.3)	6.5 (4.6–10.3)	7.4 (5.4–11.3)	0.35
Lymphocytes, ×10^9^/L	0.9 (0.6–1.6)	0.9 (0.6–1.6)	0.6 (0.4–0.9)	0.049
Neutrophils, ×10^9^/L	4.8 (3.2–7.8)	4.7 (3.1–7.6)	4.9 (4.3–7.8)	0.61
Monocytes, ×10^9^/L	0.4 (0.3–0.7)	0.4 (0.3–0.7)	0.4 (0.2–0.6)	0.45
Eosinophils, ×10^9^/L	0.0 (0.0–0.1)	0.0 (0.0–0.1)	0.0 (0.0–0.1)	0.24
Basophils, ×10^9^/L	0.0 (0.0–0.1)	0.0 (0.0–0.1)	0.0 (0.0–0.0)	0.58
Platelets, ×10^9^/L	250.5 (151.0–397.5)	248.5 (152.0–375.0)	347.0 (74.0–408.0)	0.93
Neutrophils/lymphocytes ratio	5.2 (3.4–9.0)	4.9 (3.4–8.1)	10.5 (6.9–13.4)	0.004
Platelets/lymphocytes ratio	292.1 (172.3–450.0)	284.5 (157.6–395.2)	485.5 (286.1–678.2)	0.027
C-reactive protein, mg/dL	73.8 (23.0–156.8)	72.5 (18.8–134.1)	168.7 (81.4–219.0)	0.019
Fibrinogen, mg/dL	473.0 (276.5–598.5)	469.0 (270.0–597.0)	500.0 (400.0–689.0)	0.62
D-Dimer, ng/mL	660.0 (282.0–1655.0)	622.0 (280.5–1477.0)	2000.0 (642.0–4675.0)	0.100
INR	1.2 (1.0–1.3)	1.2 (1.0–1.3)	1.2 (1.1–1.2)	0.46

*MPN* myeloproliferative neoplasms, *ET* essential thrombocythemia, *PV* polycythemia vera, *MF* myelofibrosis, *pre-PMF* prefibrotic myelofibrosis, *ASA* acetylsalicylic acid, *ICU* intensive care unit, *COPD* chronic obstructive pulmonary disease, *ACE* angiotensin-converting-enzyme, *ARBs* angiotensin II receptor blockers, *O*_2_ oxygen, *LMWH* low molecular weight heparin, *INR* international normalized ratio, IQR interquartile range.

^a^Data refer to the last follow-up of MPN control before COVID-19 diagnosis, performed at a median of 47 days earlier (IQR: 28–67).

^b^Data available for 46 of 73 patients treated with Hydroxyurea.

Figure 1c illustrates the remarkable difference in the CIF of thrombosis in patients with ET compared with the other MPN phenotypes. Of note is the rapid steepness of the curve starting from the first hours after hospitalization and reaching the peak after 30 days.

Such variables as history of thrombosis or bleeding, splenomegaly, duration of MPN disease, or proportion of patients treated with cytoreductive therapy were similar in the two groups (Table 3). Likewise, COVID-19-related symptoms were registered with comparable frequency and severity in the two groups, and no significant difference was detected in the frequency of comorbidities.

**Fig. 2:**
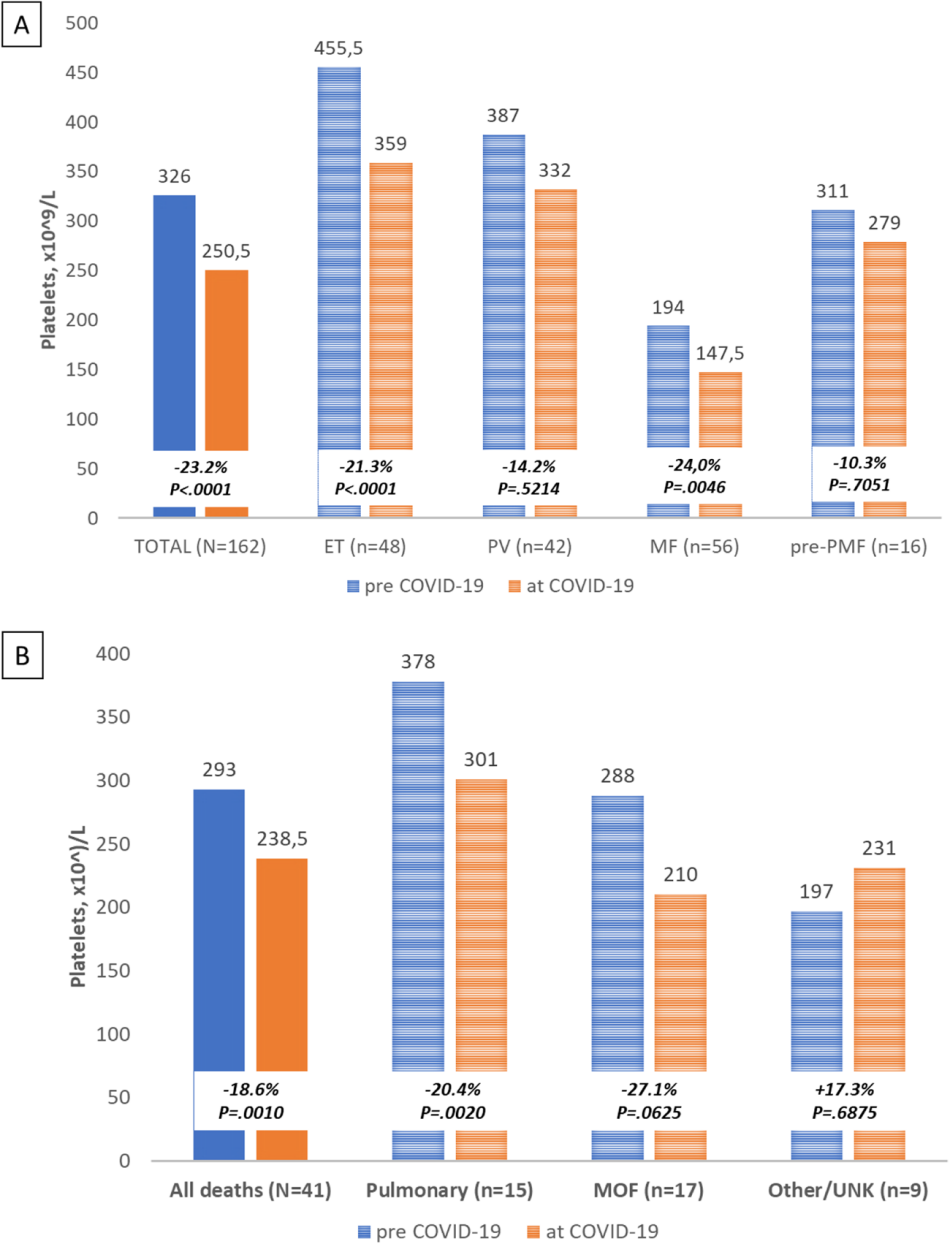
Platelet changes at COVID-19. Median platelet counts before and at COVID-19 diagnosis, by MPN phenotype (**A**) and by causes of death (**B**).

The two groups did not differ for blood hemoglobin values, leukocyte, and platelet counts at COVID-19 diagnosis. However, at COVID-19 diagnosis, patients had significantly lower (*p* < 0.0001) platelet number (median 250.5 × 10^9^/L, IQR: 151–398) than at the time of last follow-up visit performed at median of 47 days (IQR: 28–67) before diagnosis of SARS CoV-2 infection (median 326 × 10^9^/L, IQR: 218–477). This decline was remarkable in ET (−23.3%; *p* = <0.0001) and less pronounced in PV (−16.4%; *p* = 0.1730) (Fig. 2a) and was associated with higher mortality rate (*p* = 0.0010), mainly due to pneumonia (*p* = 0.0051), which was reported in higher proportion of ET than in PV and MF. Conversely, the platelet drop was not linked to a significant difference of death for MOF (*p* = 0.06) (Fig. 2b).

Levels of inflammation markers (neutrophil/lymphocyte ratio (NLR), platelet/lymphocyte ratio (PLR) and C-reactive protein (CRP)) were significantly higher in patients with thrombosis (*p* = 0.004, 0.02, 0.019, respectively). Concerning blood coagulation tests, aPTT, prothrombin time (PT), and fibrinogen values were within the normal values, while a non-significant trend for D-Dimer increase was seen in cases with thrombosis (*p* = 0.10). However, in patients with ET and thrombosis, the levels were significantly higher (median 4675.0 ng/mL, IQR: 2590.0–10,000.0) compared to cases without thrombosis (median: 367.0 ng/mL, IQR: 200.0–884.0; *p* < 0.0001).

The effects of COVID-19 severity in patients with thrombosis were remarkable in the univariate analysis as evidenced by the significant association with the need for hospitalization in the ICU, the elevated systemic inflammatory status (NLR) and the more frequent prescription of corticosteroids. The final multivariate Fine and Gray’s regression model showed that risk factors independently associated with thrombosis were transfered to ICU (sub-distribution hazard ratio [SHR] = 3.73, 95% CI = 1.14–12.23, *p* = 0.029), NLR (SHR = 1.16, 95% CI = 1.06–1.27, *p* = 0.001) and ET phenotype (SHR = 4.37, 95% CI = 1.51–12.64, *p* = 0.006), the latter being the factor with the highest SHR.

## Discussion

The primary goal of this retrospective observational study was to evaluate the rate and risk factors for thrombosis and bleeding in patients with MPN and COVID-19, collected in Europe during the first wave of pandemic, from 15 February to 30 June 2020.

### Rate of thrombosis and bleeding

Overall, the cumulative rate of arterial and VTE events was 8.6%, occurring in 14 out of 162 MPN patients during 60 days of observation for COVID-19. Most of the events during SARS-CoV-2 infection were VTE, as already reported^18,19^, indicating that their occurrence was more closely linked to infection rather than MPN, in which events are more commonly represented by arterial thrombosis^10–12^. These complications occurred in all patients but one, despite LMWH prophylaxis. In this regard, it is relevant to note that prescription of antithrombotic prophylaxis in COVID-19 patients, either managed at home or in regular wards during the first months of pandemic, was not considered a standard therapy, and this may explain why only 57% of MPN cases received antithrombotic prophylaxis which, in almost all patients, was LMWH. The dosage of this drug was heterogeneous (in 80% of patients was low-intermediate and in 20% therapeutic), mirroring the clinical practice in non-MPN patients with COVID-19^20^. The Scientific and Standardization Committee of the International Society on Thrombosis and Hemostasis (ISTH) has suggested a universal strategy of routine thromboprophylaxis with LMWH after a careful assessment of the bleeding risk^21^. However, there is a lack of studies documenting which dose of LMWH has a most favorable risk/efficacy profile; therefore, in the current clinical practice, the dose is chosen on an empirical basis, evaluating the underlying pathology concomitant with COVID19 and the presence of generic vascular risk factors^21^.

In patients with PV and MF, the rate of VTE was 4.8% and 5.4%, respectively, overall similar to that reported in non-MPN acutely ill medical patients with COVID-19 admitted in regular wards^6^. Conversely, the rate of thrombosis was higher in ET, even though the indicators of COVID-19 severity did not show differences compared with the other MPNs. In these patients, vascular events happened shortly after admission to hospital as documented by the steepness of the cumulative incidence curve that reached 16.7% after 30 days of observation. This incidence is higher than that reported in most series of non-MPN patients with COVID-19 admitted in regular ward^6^. We point out that the higher frequency of VTE, in comparison with arterial thrombosis, is a finding strictly linked to the infection and contrasts with the experience outside COVID-19 settings, in which the opposite is the rule^10–12^. Remarkably, these events had a notable impact on survival of these patients that was around 25% inferior than in ET patients without thrombosis.

As expected, patients transferred to ICU (*n* = 17/162; 10.6%) experienced a significantly higher incidence of thrombosis than those requiring non-invasive respiratory support and admitted in regular wards (*p* = 0.004).

Unfortunately, we could not confirm the relationship between D-Dimer elevation and VTE^6,22^, likely due to the limited number of tests carried out in most of these patients; only a trend for this association was shown (*p* = 0.10). However, in ET patients with VTE, the D-Dimer values were significantly higher than in the ones without thrombosis (*p* < 0.0001).

While the peak of thrombotic events occurred in the first days after COVID-19 diagnosis, bleeding episodes were reported later, starting 2 weeks afterwards, and accounted for a cumulative rate of 5%, a figure higher than 2.3% reported in the non-ICU general population with COVID-19^23^. We underscore the severity of these events, requiring blood transfusion particularly in MF. In 4 of our cases, a coagulopathy or severe thrombocytopenia were associated with severe bleeding suggesting caution on the empiric intensification of LMWH particularly in MF.

### Risk factors

The low number of bleeding events precluded an analysis of risk factors and also in multivariate models for thrombosis, few confounders could be included given that only 14 events were ascertained. As expected, patient transfer to ICU and NLR inflammatory marker were confirmed significant factors associated with total thrombosis.

Of great interest is the association between ET and thrombosis, regardless of inflammation indicators and the severity of COVID-19. This finding requires further confirmation in a larger series of patients; however, it highlights the role of platelets in the thrombogenesis of MPN patients with COVID-19. During the acute phase of infection, our patients had platelet count higher than the other MPN but significantly lower than the value collected in the last follow-up before the infection, possibly indicating a platelet consumption due to low grade disseminated coagulation^24^. This process might be related to the systemic endothelial vascular cell damage associated with viral infection^4,5^ and involve the interaction of platelets, leukocytes, neutrophil extracellular trap (NET) formation^25^, and vascular endothelium, leading to classic arterial and venous thrombosis, but also to lung and other tissues vessel occlusion and hypoxia^1,2^. We can speculate that in at least some of the MPN patients who died for sudden respiratory deterioration, the decline of platelet number associated with the initial diagnosis of pneumonia might be due to pulmonary thrombosis, in the context of a “local” intravascular coagulation and complement^26^.

### Limitations and conclusion

The main limitations of this observational study are related to its retrospective design and to the limited number of patients. However, this constrain can hardly be avoided when dealing with both a rare condition, such as MPNs, and an exceptional situation of emergency, such as the COVID-19 crisis.

In conclusion, our study should be considered as an exploratory analysis whose results pave the way for larger follow-up studies hopefully not having the same limitations. In particular, the enhanced susceptibility to ET-associated thrombosis is a finding that needs to be delved, especially for the possibility to tailor antiplatelet and/or antithrombotic drugs in SARS CoV-2-infected patients who develop COVID-19.

## Supplementary information

Supplementary material
