# Supplementary material for: Among classic myeloproliferative neoplasms, essential thrombocythemia is associated with the greatest risk of venous thromboembolism during COVID-19
Source: Blood Cancer J. 2021 Feb 4;11(2):21. doi: 10.1038/s41408-021-00417-3 (PMC7871138; doi:10.1038/s41408-021-00417-3)
Supplement: Supplementary file 1 — Supplementary material [file 41408_2021_417_MOESM1_ESM.pdf]

Supplementary information

Table 1S. MPN and COVID-19 characteristics, by ET vs. other MPN phenotypes

	Total N=162	ET N=48	Other MPNs N=114	p-value
**Sex, n (%)**				0.29
Female	66 (40.7%)	23 (47.9%)	43 (37.7%)	
Male	96 (59.3%)	25 (52.1%)	71 (62.3%)	
**Age (years), median (IQR)**	70.6 (60.0-79.9)	71.6 (53.9-80.3)	70.3 (60.9-79.7)	0.70
<60 years, n (%)	40 (24.8%)	15 (31.3%)	25 (22.1%)	0.35
60-70 years, n (%)	36 (22.4%)	8 (16.7%)	28 (24.8%)	
>70 years, n (%)	85 (52.8%)	25 (52.1%)	60 (53.1%)	
**JAK2V617F, n (%)**	112 (70.9%)	27 (57.4%)	85 (76.6%)	0.016
**CALR, n (%)**	26 (29.5%)	13 (39.4%)	13 (23.6%)	0.12
**MPL, n (%)**	5 (6.0%)	2 (6.9%)	3 (5.6%)	0.81
**Previous thrombosis, n (%)**	23 (14.4%)	8 (16.7%)	15 (13.4%)	0.59
**Previous bleeding, n (%)**	12 (7.5%)	4 (8.3%)	8 (7.1%)	0.79
**MPN duration before COVID-19 onset (years), median (IQR)**	5.9 (2.9-10.8)	6.9 (4.5-15.2)	5.4 (2.1-9.7)	0.010
**Blood values before COVID-19 diagnosis*, median (IQR)**				
Hemoglobin, g/dL	13.0 (11.4-14.2)	13.1 (12.1-14.2)	12.6 (10.9-14.2)	0.056
White blood cells, x10^9/L	7.2 (5.4-10.3)	7.1 (5.5-8.2)	7.3 (5.3-11.3)	0.61
Platelets, x10^9/L	326.0 (218.0-477.0)	455.5 (354.0-550.0)	275.0 (164.0-402.0)	<0.001
**MPN-directed therapy before COVID-19 diagnosis*, n (%)**				
Hydroxyurea	73 (45.1%)	29 (60.4%)	44 (38.6%)	0.011
Ruxolitinib	40 (24.7%)	2 (4.2%)	38 (33.3%)	<0.001
Anagrelide	8 (4.9%)	8 (16.7%)	0 (0.0%)	<0.001
Interferon	4 (2.5%)	0 (0.0%)	4 (3.5%)	0.19
Other cytoreductive drugs	5 (3.1%)	0 (0.0%)	5 (4.4%)	0.14
ASA	94 (58.0%)	32 (66.7%)	62 (54.4%)	0.15
**Patient disposition, n (%)**				0.96
Home	40 (24.8%)	11 (22.9%)	29 (25.7%)	
Regular ward	104 (64.6%)	32 (66.7%)	72 (63.7%)	
ICU	17 (10.6%)	5 (10.4%)	12 (10.6%)	
**Main symptoms, n (%)**				
Fever	130 (80.2%)	41 (85.4%)	89 (78.1%)	0.28
Dispnea	89 (54.9%)	29 (60.4%)	60 (52.6%)	0.36
Gastrointestinal	19 (11.7%)	4 (8.3%)	15 (13.2%)	0.38
**Comorbidities, n (%)**				
Cerebrovascular disease	21 (13.0%)	9 (19.1%)	12 (10.5%)	0.14
Chronic dialysis/Kidney disease	15 (9.3%)	4 (8.5%)	11 (9.6%)	0.82

Chronic heart failure	22 (13.8%)	6 (12.8%)	16 (14.2%)	0.82
COPD	22 (13.7%)	7 (14.9%)	15 (13.2%)	0.77
Current/former tobacco smoker	33 (23.2%)	12 (28.6%)	21 (21.0%)	0.33
Hyperlipidemia	45 (29.0%)	12 (26.1%)	33 (30.3%)	0.60
Hypertension	97 (61.4%)	32 (68.1%)	65 (58.6%)	0.26
Antihypertensives use	93 (60.8%)	29 (61.7%)	64 (60.4%)	0.88
ACE inhibitors/ARBs	54 (36.7%)	22 (46.8%)	32 (32.0%)	0.082
Other	33 (21.6%)	7 (14.9%)	26 (24.5%)	0.18
Diabetes mellitus	19 (11.9%)	4 (8.5%)	15 (13.4%)	0.39
**O_2 saturation (%), median (IQR)**	93.0 (88.0-96.0)	93.0 (91.5-95.0)	92.0 (88.0-97.0)	0.73
**COVID-19-directed drugs, n (%)**				
Steroid	41 (27.2%)	9 (19.1%)	32 (30.8%)	0.14
Antibiotic	104 (68.9%)	35 (74.5%)	69 (66.3%)	0.32
Hydroxychloroquine	93 (59.6%)	32 (66.7%)	61 (56.5%)	0.23
Antiviral	54 (35.1%)	17 (36.2%)	37 (34.6%)	0.85
Lopinavir/Ritonavir	44 (88.0%)	14 (87.5%)	30 (88.2%)	1.00
Other	6 (12.0%)	2 (12.5%)	4 (11.8%)	
Experimental	17 (10.7%)	3 (6.3%)	14 (12.6%)	0.23
Tocilizumab	13 (76.5%)	3 (100.0%)	10 (71.4%)	1.00
Ruxolitinib	2 (11.8%)	0 (0.0%)	2 (14.3%)	
Other	2 (11.8%)	0 (0.0%)	2 (14.3%)	
Antithrombotic	88 (56.8%)	30 (62.5%)	58 (54.2%)	0.34
LMWH	84 (54.2%)	30 (62.5%)	54 (50.5%)	0.16
Low-intermediate dose	62 (79.5%)	24 (82.8%)	38 (77.6%)	0.16
Therapeutic dose	16 (20.5%)	5 (17.2%)	11 (22.4%)	
**Laboratory parameters at COVID-19 diagnosis, median (IQR)**				
Hemoglobin, g/dL	12.4 (10.0-13.5)	12.9 (12.0-14.1)	11.5 (9.4-13.4)	0.006
White blood cells, x10^9/L	6.6 (4.7-10.3)	6.5 (4.7-9.2)	6.7 (4.7-10.5)	0.73
Lymphocytes, x10^9/L	0.9 (0.6-1.6)	1.0 (0.6-1.6)	0.8 (0.5-1.6)	0.29
Neutrophils, x10^9/L	4.8 (3.2-7.8)	4.6 (2.5-6.8)	4.9 (3.4-7.9)	0.54
Monocytes, x10^9/L	0.4 (0.3-0.7)	0.4 (0.3-0.5)	0.4 (0.3-0.7)	0.41
Eosinophils, x10^9/L	0.0 (0.0-0.1)	0.0 (0.0-0.1)	0.0 (0.0-0.1)	0.47
Basophils, x10^9/L	0.0 (0.0-0.1)	0.0 (0.0-0.0)	0.0 (0.0-0.1)	0.13
Platelets, x10^9/L	250.5 (151.0-397.5)	359.0 (208.0-458.0)	229.0 (121.0-328.0)	0.012
Neutrophils/lymphocytes ratio	5.2 (3.4-9.0)	4.6 (3.0-7.4)	5.6 (3.6-9.9)	0.11
Platelets/lymphocytes ratio	292.1 (172.3-450.0)	306.6 (248.7-457.8)	276.4 (147.4-414.0)	0.23
C-Reactive Protein, mg/dL	73.8 (23.0-156.8)	72.5 (31.9-161.5)	75.0 (17.5-148.5)	0.71
Fibrinogen, mg/dL	473.0 (276.5-598.5)	422.0 (267.0-529.0)	499.0 (330.0-617.0)	0.26
D-Dimer, ng/mL	660.0 (282.0-1655.0)	458.5 (223.5-2008.5)	789.0 (337.0-1504.0)	0.72
INR	1.2 (1.0-1.3)	1.1 (1.0-1.3)	1.2 (1.1-1.3)	0.52

** Data refer to the last follow-up of MPN control before COVID-19 diagnosis, performed at a median of 47 days earlier (IQR: 28-67).*

Abbreviations. MPN=myeloproliferative neoplasms; ET=essential thrombocythemia; PV=polycythemia vera; MF=myelofibrosis; pre-PMF=prefibrotic myelofibrosis; ASA=acetylsalicylic acid; ICU=intensive care unit; COPD=chronic obstructive pulmonary disease; ACE=angiotensin-converting-enzyme; ARBs=angiotensin II receptor blockers; O_2=oxygen; LMWH=low molecular weight heparin; INR=international normalized ratio; IQR=interquartile range.

Figure 1S. Kaplan-Meier survival curves, by thrombosis

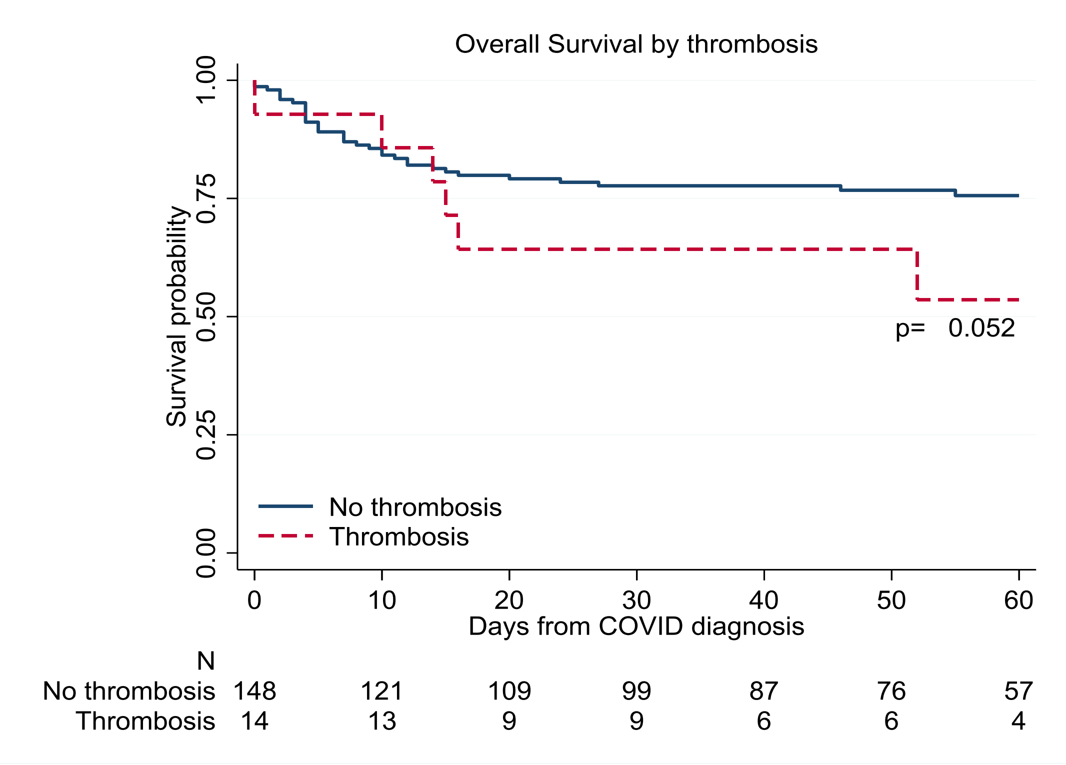

Legend. P-values calculated by log-rank test.
